# Root-System Architectures of Two Cuban Rice Cultivars with Salt Stress at Early Development Stages

**DOI:** 10.3390/plants10061194

**Published:** 2021-06-11

**Authors:** Alenna Vázquez-Glaría, Bettina Eichler-Löbermann, F. G. Loiret, Eduardo Ortega, Mareike Kavka

**Affiliations:** 1 Laboratorio de Fisiología Vegetal, Dpto. Biología Vegetal, Facultad de Biología, Universidad de La Habana, Avenida de la Universidad s/n entre Ronda y G, CP Habana 10400, Cuba; alenna@fbio.uh.cu (A.V.-G.); loiret@fq.uh.cu (F.G.L.); eortega@fq.uh.cu (E.O.); 2 Agronomy, Faculty of Agriculture and Environmental Sciences, University of Rostock, Justus-von-Liebig-Weg 6, 18059 Rostock, Germany; bettina.eichler@uni-rostock.de

**Keywords:** *Oryza sativa*, root-system architecture, salt stress, phosphorus deprivation, epibrassinolide

## Abstract

Soil salinity is a critical problem for rice production and is also often associated with phosphors (P) deficiency. Plant hormones, like brassinosteroids, were shown to play a role in plant responses to different stresses and are also expected to mitigate salt stress. The aim of this study was to compare shoot growth and root architecture traits of two rice cultivars (INCA LP-5 and Perla de Cuba) during early plant development in response to salt, P limitation and a brassinosteroid. Seeds were placed in (I) paper rolls for 7 days and (II) mini-rhizotrons for 21 days without or with salt (50 mM NaCl), without or with 24-epibrassinolide (10^−6^ M) pre-treatment, and with two levels of P (10 or 1 ppm). The root system of LP-5 was larger in size and extent, while the roots of Perla were growing denser. Salt affected mainly the size- and extent-related root characteristics and explained about 70% of the variance. The effect of P was more pronounced without salt treatment. In Perla, P supply reduced the salt effect on root growth. The brassinosteroid had hardly any effect on the development of the plants in both experiments. Due to the high dependence on experimental factors, root length and related traits can be recommended for selecting young rice cultivars regarding salt stress and P deprivation.

## 1. Introduction

Soil salinity is a critical problem for plant growth and productivity [1]. About 830 million ha of land worldwide are affected by salinity, about half of it being in Asia [2]. In Cuba, about 15% of the agricultural area and about 9% of the surface of the country are affected by salts [3]. The effects of salinity on plant growth are associated with the low osmotic potential of the soil solution, leading to osmotic, ionic and oxidative stress, as well as nutritional imbalances [4,5,6,7].

Rice (*Oryza sativa* L.) is a major food crop of the developing world and is often cultivated on salt-affected soils [8,9]. Although rice can tolerate a small amount of saltwater without compromising the yield [10], under stronger salinity, rice plants show several morphological, physiological and biochemical alterations [11,12,13]. The main effects are the reduction of water uptake and disturbance of nutrient uptake, especially of phosphorus (P) [10]. In saline soils, phosphate ions tend to form hardly soluble precipitates, which are not available to plants, leading to the combination of salt stress and P starvation [14,15].

Roots link the growth medium with the aboveground plant parts and are essential for water and nutrient uptake from the soil. Usually, salinity inhibits root growth, although the extent is strongly species-dependent [16].

Salt-induced decreases in the root systems of different plants like *Chenopodium quinoa* [17], *Medicago truncatula* [18] and *Zea mays* [19] have been described. Studies regarding the root growth of rice under salt stress showed a reduction of the root length [20], reduced depth of the rooting system and lower root weight [11,12]. Many of these studies included different genotypes and showed that salt effects on roots vary not only between species but also between genotypes within one species. Salt stress often inhibits root growth more than shoot growth, which reduces the root-to-shoot ratio [12]. On the other hand, plants often increase the root-to-shoot ratio in response to P depletion to acquire P more efficiently [21]. Thus, shoots and roots exhibited different responses to salinity and P deficiency, with more marked effects of salinity on roots and of P deficiency on shoots [22].

The term root-system architecture (RSA) includes aspects of root anatomy and morphology, as well as topology and distribution [23,24]. For rice, the development of lateral roots in particular was described in relation to salt stress. According to Krishnamurthy et al. [25], salt stress increased the number of lateral roots. In more detail, Toyofuku et al. [26] showed that mainly the L-type lateral roots were enhanced under salt stress, while crown roots and S-type lateral roots were decreased.

Phosphorus deficiency may influence the RSA in a different way than salt stress [22]. In rice plants, P shortage reduced the number of nodal roots as well as lateral root length and density, with the magnitude of the response being genotype-dependent [27]. Because P availability is highest in topsoils, more shallow root systems increase P acquisition [28].

Studies regarding the effects of combined stress of salt and P shortage on roots exist for barley (*Hordeum vulgare*) [29,30], maize [22] and Arabidopsis [31]. They show that the responses to one factor can overrule the responses to the other depending on strength of the factor, measured parameters and genotype. Studies of the rice RSA in response to a combination of salt stress and low P are still missing to our knowledge.

Growth and development of the root system require a coordinated regulation, which is closely linked to phytohormones. Brassinosteroids (BRs) are steroidal hormones that play an essential role in many aspects of plant growth and have effects on plant responses to different stresses [32,33]. BRs, in low concentrations, promote root growth through the control of root meristem size and cell elongation [34,35,36,37] and promote lateral root initiation [38]. Moreover, treatments of rice seeds with BRs may also mitigate the effects of salinity by promoting germination and growth in presence of sodium chloride [39,40] and alleviating oxidative damage under saline stress [41]. Studies on the potential of BRs to mitigate P starvation stress or the combined stress induced by salinity and low P availability are still missing.

The selection of genotypes according to their root system is a feasible strategy to increase yields in crop production [42]. Under field or pot conditions, the complete excavation of a root system is almost impossible with risks of root structure loss. In order to better access the root system, investigations with roots in PVC pipes, hydroponic systems or rhizotrons were carried out [43], and according to Shrestha et al. [44], rhizotrons are the preferred method for rice root screening, particularly since root angles can be assessed. According to Ali et al. [45] and Rasel et al. [46], seedling stage screening is suitable to determine salt-tolerant genotypes because variations in the genotypes on this stage are genetically controlled.

The aim of this study was to compare the effects of salt stress and seed pre-treatment of BRs on the RSA in two Cuban rice cultivars differing regarding the optimal sowing period (INCA LP-5 and Perla de Cuba) at seedling stage and, in combination with reduced P, in young plants. The INCA LP-5 cultivar is characterized by very vigorous material with optimal sowing in the dry season from December to February [47]. In contrast, Perla de Cuba’s optimum sowing period is in the wet season from January to July. We used two experimental designs with paper rolls and mini-rhizotrons for the measurement of RSA traits. Based on outcomes of previous studies, we expected (I) that salt stress reduces root and shoot weight and affects the RSA in seedlings and young plants, (II) that different P and BR supply modify salt stress impacts and (III) that cultivars respond to the treatments differently. The results could provide knowledge for breeding trait selection and further mitigation measures.

## 2. Materials and Methods

### 2.1. Plant Material and Seed Pre-Treatment

For all experiments, the two *Oryza sativa* L. subsp. *indica* cultivars INCA LP-5 (LP-5) of the National Institute of Agricultural Sciences, San José de las Lajas, Cuba [48] and Perla de Cuba (Perla), produced by the Grain Research Institute, San Antonio de los Baños, Cuba, were used. Prior to germination, seeds were embedded in water or BR solution (10^−6^ M 24-Epibrassinolide, Sigma-Aldrich, St. Louis, MO, USA) for 14 h at 100 rpm.

### 2.2. Roll Experiment

After pre-treatment with water or BR, three seeds of one genotype and treatment were placed on one cellulose filter paper (Type 14a, Carl Roth, Karlsruhe, Germany) piece of 25 cm × 16.6 cm with 3 cm between each and 2 cm below the top. Each filter paper was rolled to make a cigar roll and moistened with dH_2_O or 50 mM NaCl. Every treatment combination (cultivar × salt × BR) was replicated five times, amounting to 40 rolls and 120 plants (three plants per roll). Every treatment combination was placed in a separate plastic bag to keep the humidity (in a total of eight bags). The rolls were incubated vertically at 28 °C in the dark for seven days. At harvest for each roll, root fresh weight and shoot fresh weight were measured and divided by three to gain the average weight per plant. For each individual plant, a root image was acquired.

### 2.3. Mini-Rhizotron Experiment

Plants were grown from October to November 2019 in a greenhouse at the Faculty of Agriculture and Environmental Sciences, University of Rostock, Germany. The temperature was 26/21 °C (day/night), and the light intensity was ~7500 Lux. After pre-treatment, four seeds of each cultivar were placed in mini-rhizotrons [49] for germination. The mini-rhizotrons were randomly settled inside plastic boxes with an angle of ~70°. The boxes were filled with four different modified Yoshida solutions [50]: original Yoshida solution with NaH_2_PO_4_ at a P concentration of 10 ppm and no salt, Yoshida solution with a low concentration of P (1 ppm), Yoshida solution with salt (50 mM NaCl) and Yoshida solution with a low concentration of P (1 ppm) and salt (50 mM NaCl). For germination, the boxes were covered with aluminum foil during the first two days. Three of the four seedlings were removed after three days to grow one plant per mini-rhizotron. The experiment was conducted with five replications per treatment (salt × P × BR) with each cultivar for 21 days. The nutrient solution was changed every week. Roots were protected from light by covering the boxes with aluminum foil, leaving an opening where the seedlings were. At harvest root and shoot fresh weight were measured, and a root image was acquired.

### 2.4. Root Image Acquisition and Analysis

Roots from rolls and mini-rhizotrons were carefully scanned without disturbing the root system (CanoScan LiDE 210, Canon, Krefeld, Germany). Image resolution was set to 300 dpi and a black background was used to maximize the contrast. Image processing and phenotyping were carried out with two free software packages for RSA phenotyping: GiA Roots [51] was used to analyze nineteen parameters describing the size, extent, shape and distribution of the whole root network. The ImageJ [52] plugin SmartRoot [53] was used to analyze the root angle between the two outmost mesocotyl roots in the rhizotrons, primary root length and number of lateral roots along the entire primary root in the rolls experiment, as well as lateral root density. Lateral root density was measured along the entire primary root for seedlings grown in rolls. For seedlings grown in mini-rhizotrons, lateral root density was measured along the three cm before the last lateral root on one seminal root. All traits, including the units and abbreviations, are listed in Table 1.

### 2.5. Plant P Concentration

Concentration of P in the plants from the mini-rhizotrons was measured after drying at 60 °C for six days and weighing. Due to very little biomass in some treatments, roots and shoots of each plant were burned together in a muffle furnace at 550 °C for 4 h. Digestion was carried out in 25% HCl according to Page et al. [54]. Element concentrations were measured using an Optima 8300 DV ICP-OES spectrometer (Perkin Elmer, Waltham, MA, USA). The P concentration was estimated as mg P per g plant dry matter.

### 2.6. Statistical Analyses

R (version 3.6.2; [55]) was used for statistical analyses. A linear model with the factors NaCl and BR treatment and their interactions was fitted for the roll experiment and with the factors NaCl, P and BR treatment, and their interactions for the rhizotron experiment and an analysis of variance (ANOVA) was calculated. Furthermore, an ANOVA was applied to check for significant differences between the cultivars within the treatment combinations. To calculate the proportion of variance explained by single factors, a type-III ANOVA using the package “car” [56] was calculated. The sum of squares of all factors, factor interactions and residuals were added up, and the proportion of each calculated. For the radar charts, the R library “fmsb” [57] was used with z-scored data over all treatments and both cultivars.

## 3. Results

### 3.1. Roll Experiment

**Figure 1:**
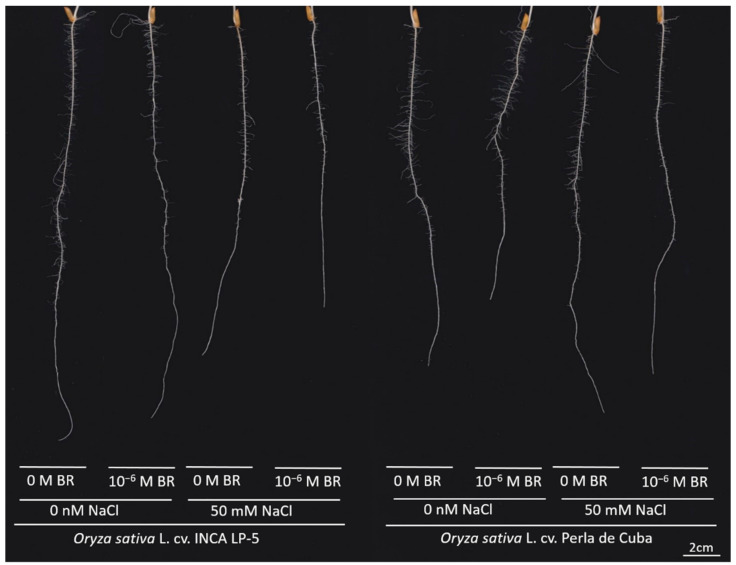
Representative root images of the two rice cultivars INCA LP-5 and Perla de Cuba after 7 days of growth in rolls with or without salt (NaCl) and 24-epibrassinolide (BR) pre-treatment.

The two rice cultivars LP-5 and Perla developed a primary root with first-order lateral roots within seven days of growth in cigar rolls (Figure 1). Although the shoot weight was higher in LP-5 than in Perla without salt, there was no difference in root weight between both cultivars (Table 2). LP-5 reduced shoot and root weight under salt stress conditions to 67% and 59%, respectively. Perla maintained shoot and root growth under salt stress. The BR treatment had no significant effect on root and shoot weight. Root-to-shoot ratio did not change significantly when plants were treated with salt (Table 2). In the absence of salt stress, the primary root length was higher in LP-5 than in Perla (Table 2). While LP-5 reduced the primary root length under NaCl stress, Perla maintained the primary root length. The BR treatment reduced the primary root length in Perla. The lateral root density decreased under salt stress in both cultivars (*p* < 0.01). The total network length was higher in LP-5 than in Perla without salt and showed a strong reduction under salt stress of about 50% in LP-5. Perla maintained the network length under salt stress. A pre-treatment with BR resulted in a lower network length in Perla (Table 2).

### 3.2. Mini-Rhizotron Experiment–Biomass and P Concentration

**Figure 2:**
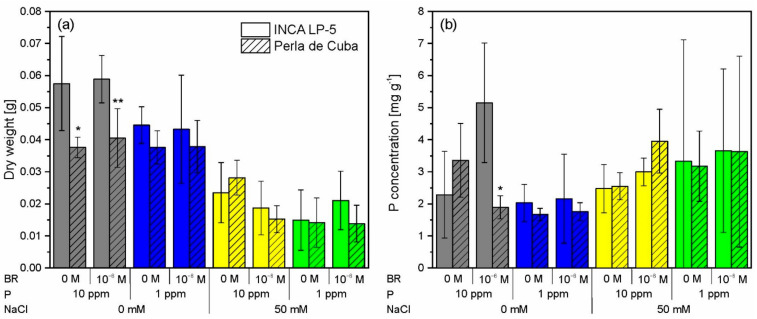
Dry weight (**a**) and phosphorus (P) concentration (**b**) of the two rice cultivars INCA LP-5 and Perla de Cuba after growth for three weeks in mini-rhizotrons treated with P depletion, NaCl or both and pre-treated with 24-epibrassinolide (BR). Means ± SD (n = 5). Differences between P (*p* < 0.05) and NaCl (*p* < 0.001) treatments were significant for dry weight in both cultivars; differences between NaCl treatments were significant for P concentration of Perla de Cuba (*p* < 0.01, ANOVA). Significant differences between cultivars in each treatment are indicated with asterisks (*p* < 0.05 * 0.01 **).

After three weeks of growth in mini-rhizotrons, the plant biomass was reduced upon P deprivation (*p*_P_ < 0.05) and NaCl treatment (*p*_NaCl_ < 0.001) in both LP-5 and Perla cultivars (Figure 2a). Salt stress reduced the biomass of LP-5 (to about 36% of the control) more than the biomass of Perla (to 56% of the control). The larger cultivar LP-5 reduced the biomass in reaction to P deprivation from 58 mg to 44 mg, while Perla did not react to P deprivation alone. Double treatment of low P and salt reduced the biomass only slightly more than salt stress alone. The BR pre-treatment showed no significant effect on the biomass, although it was slightly lower with BR under salt stress in both cultivars (Figure 2a).

**Figure A1:**
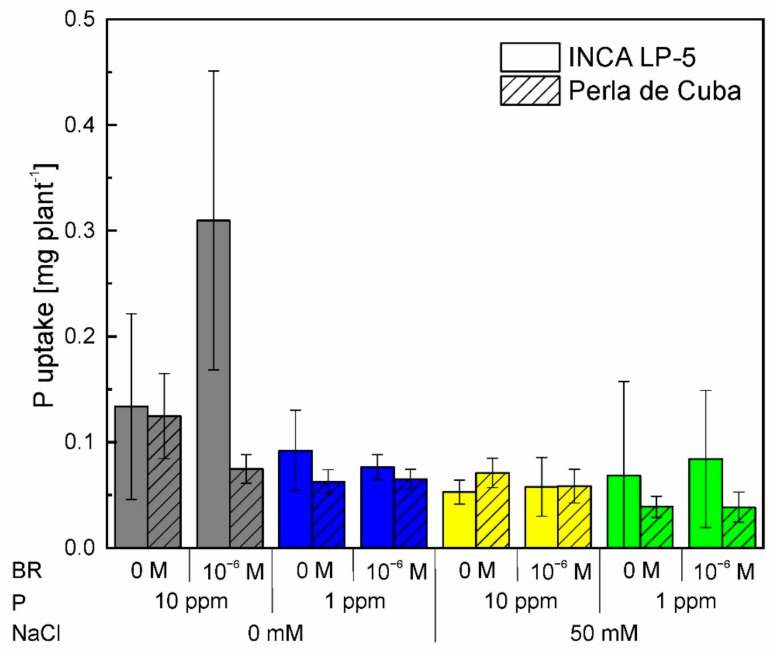
Phosphorus uptake of the two rice cultivars INCA LP-5 and Perla de Cuba after growth for three weeks in mini-rhizotrons treated with phosphorus (P) depletion, NaCl or both and pre-treated with 24-epibrassinolide (BR). Mean ± SD, n = 5.

Plant P concentration was higher in plants with salt stress than without salt stress (*p*_NaCl_ > 0.05 in LP-5, *p*_NaCl_ < 0.01 for Perla), although the variation was very high within the salt-treated groups (Figure 2b) because of the small amount of plant biomass. Due to the reduced growth, the total plant P uptake was lower in plants treated with salt than without salt stress (Figure A1). In both cultivars, the P concentration was reduced at P depletion in the absence of salt stress (*p*_P_ > 0.05). BR increased P concentration in LP-5 and decreased it in Perla in the treatments without salt and with high P. In the salt-treated groups, the P concentration was slightly higher with BR treatment than without BR treatment (Figure 2b).

### 3.3. Root-System Traits in Mini-Rhizotrons

Twenty-two root-system traits of the two rice cultivars LP-5 and Perla were evaluated after three weeks of growth in mini-rhizotrons. The traits were divided into the categories “size”, “distribution”, “extent” and “shape” according to Topp et al. [58] (Table 1).

**Figure 3:**
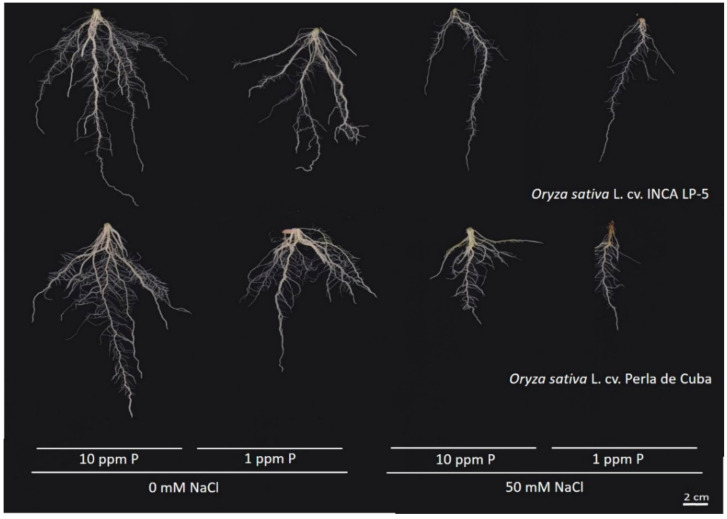
Representative root images of the two rice cultivars INCA LP-5 and Perla de Cuba after three weeks growth in mini-rhizotrons treated with low phosphorus (P), high salt (NaCl) or both.

In general, the root system of LP-5 was larger in size and extent, with the roots of Perla growing denser (Figure 3).

**Figure 4:**
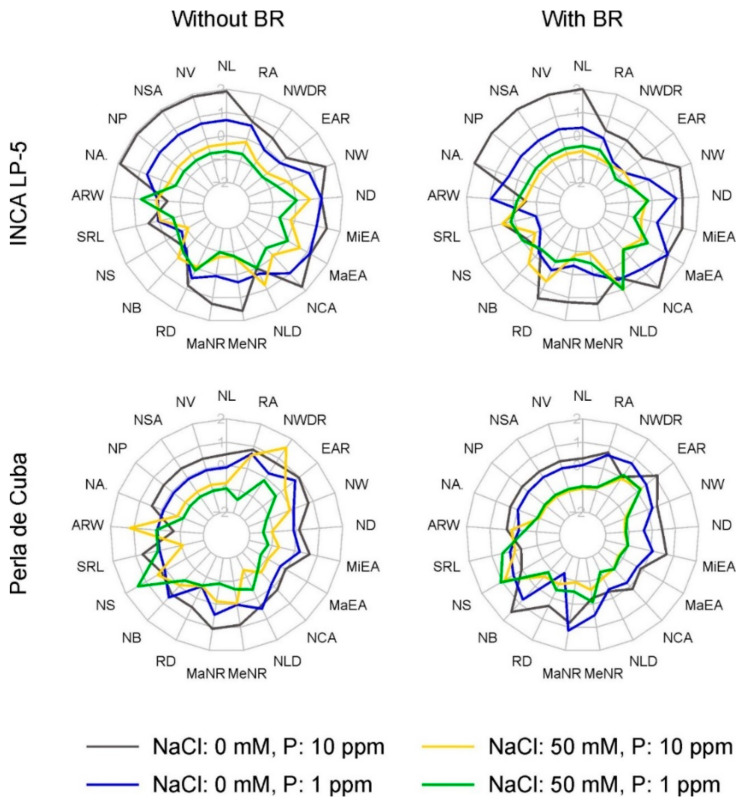
Radar chart of root-system traits (z-score transformation) of the two rice genotypes INCA LP-5 and Perla de Cuba pre-treated with or without 24-epibrassinolide (BR). Grey: control, blue: low P, yellow: high NaCl, green: low P and high NaCl. NL: network length; NV: network volume; NSA: network surface area; NP: network perimeter; NA: network area; ARW: average root width; SRL: specific root length; NS: network solidity; NB: network bushiness; RD: lateral root density; MaNR: maximum number of roots; MeNR: median number of roots; NLD: network length distribution; NCA: network convex area; MaEA: major ellipse axis; MiEA: minor ellipse axis; ND: network depth; NW: network width; EAR: ellipse axis ratio; NWDR: network width-to-depth ratio; RA: root angle.

**Figure 5:**
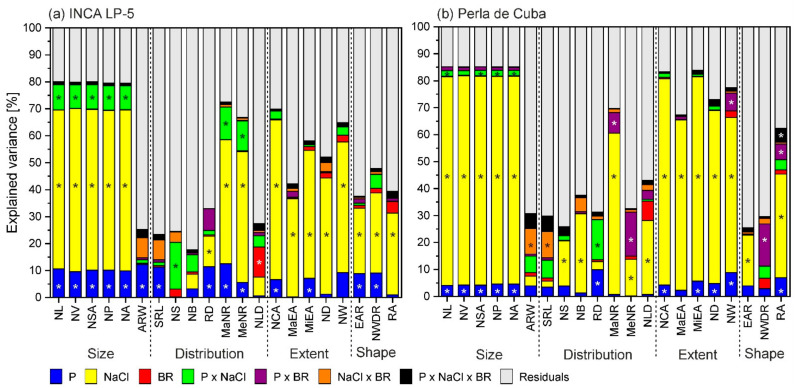
Variance in root-system traits (%) explained by the experimental factors phosphorus (P), salt (NaCl), 24-epibrassinolide (BR) and their interactions in the two rice genotypes INCA LP-5 (**a**) and Perla de Cuba (**b**). Significant factors are marked with an asterisk. NL: network length; NV: network volume; NSA: network surface area; NP: network perimeter; NA: network area; ARW: average root width; SRL: specific root length; NS: network solidity; NB: network bushiness; RD: lateral root density; MaNR: maximum number of roots; MeNR: median number of roots; NLD: network length distribution; NCA: network convex area; MaEA: major ellipse axis; MiEA: minor ellipse axis; ND: network depth; NW: network width; EAR: ellipse axis ratio; NWDR: network width-to-depth ratio; RA: root angle.

The root-system size in both cultivars was reduced with low P and high NaCl with a stronger effect of NaCl. There was hardly any additional effect of combined stress and no effect of BR treatment (Figure 4). The network length was larger in LP-5 with about 770 cm (average of without and with BR) than in Perla with 400 cm length, and these lengths were reduced to 180 cm and 85 cm, respectively, in the combined treatment (Table 3, Table A1). The closely related size parameters network volume (NV), network surface area (NSA), network perimeter (NP) and network area (NA) showed similar behavior. About 80% of the variance of all these in LP-5 and 85% in Perla were explained by the factors NaCl, P and the interaction between P and NaCl, with a dominant influence of NaCl (about 60% in LP-5 and 75% in Perla) (Figure 5).

The parameters describing the distribution of the roots within the root system are not so closely related among themselves and showed more diverse reactions in response to stress. Here, the influence of NaCl and P on the variance of the traits was in general high in LP-5. In Perla, BR treatment significantly influenced the maximum number of roots (MaNR) in interaction with P (Table 3, Table A1). The variance of most of the distribution traits was explained by less than 40% by the factors (Figure 5). The maximum and medium number of roots in LP-5 and maximum number of roots in Perla were explained mostly by NaCl. The variance in lateral root density (RD) was explained only by about 30%: by NaCl and P in LP-5 and by P and P × NaCl in Perla. The root density was reduced by NaCl and low P from 16 cm^−1^ and 14 cm^−1^ in control to 13 cm^−1^ and 12 cm^−1^ in the combined treatment in LP-5 and Perla, respectively (Table 3, Figure 4).

Network solidity (NS), standing for the density of roots, was higher for Perla than for LP-5 and increased further for Perla due to salt stress. Furthermore, network bushiness (NB) was lower in LP-5 than in Perla without salt, indicating a more homogenous branching distribution in LP-5 (Figure 4). NaCl addition increased network bushiness in LP-5 slightly and not significantly and reduced network bushiness in Perla significantly (Table 3). Most of the variance in network solidity and network bushiness was explained by P × NaCl in LP-5, whereas it was NaCl in Perla. Only about 25% of the variance was explained by all factors (Figure 5). The values for network length distribution (NLD) were higher in LP-5 when treated with BR, meaning a shift of root distribution to the depth. Perla only had significantly lower values when treated with salt (Table 3). Specific root length (SRL) as root length per root volume showed only slight responses with no clear pattern. In LP-5, the variances of specific root length and the related average root width (ARW), which showed the opposing behavior, were explained mostly by P, while in Perla, they were explained mostly by NaCl × BR (Figure 5).

The extent traits were influenced similarly to the size traits: network convex area (NCA), network depth (ND) and network width (NW) and the related major and minor ellipse axis (MaEA and MiEA) showed lower values for Perla than for LP-5 and a decrease when treated with low P or NaCl (Figure 4). A higher proportion of the variance was explained by the factors in Perla than in LP-5, with NaCl being the factor that explained most of it in both cultivars (Figure 5). The network depth was 16.5 cm and 12.4 cm for LP-5 and Perla, respectively, in control conditions, and 11.8 cm and 5.3 cm in combined treatment. The network width was reduced from 10.6 cm and 7.8 cm to 3.7 cm and 2.7 cm in LP-5 and Perla, respectively (Table 3, Table A1).

The shape parameters were less well explained than size end extent parameters (Figure 5). Here, too, the factor NaCl influenced the variance most with the exception of network width-to-depth ratio (NWDR) in Perla, which was explained most by P × BR. The network width-to-depth ratio was reduced by P without BR and increased by P with BR pre-treatment in Perla (Figure 4). In LP-5, network width-to-depth ratio was significantly reduced by P and NaCl. The root angle (RA) was reduced by NaCl (Table 3).

## 4. Discussion

### 4.1. Strong Effect of Salt Stress on Rice Growth and Root Architecture

Plant-biomass- and root-size-related parameters were affected by salt in both experiments. LP-5 developed higher biomasses and a larger root system during the experiments without salt stress but was more affected by the salt stress than Perla.

In this study, salt stress of 50 mM NaCl, corresponding roughly to an electrical conductivity (EC) of 5 dS m^−1^, was applied in both experiments, which can be considered as moderate for rice, while more than 8 dS m^−1^ is high [10].

Although considered moderate, in the rhizotron experiment, the salt stress applied reduced the plant biomass and root network length (NL) considerably by about 50 to 70% (Figure 2a, Table 3). In contrast, a relatively low reduction of plant growth of about only 20% was found in a system with rice plants exposed to much higher salt stress of 100 mM NaCl [11] or even 200 mM NaCl [13]. The relatively strong effect of 50 mM NaCl in our study can be related to the direct contact of the plants with the NaCl-containing solution during the susceptible phase of early plant development.

**Figure A2:**
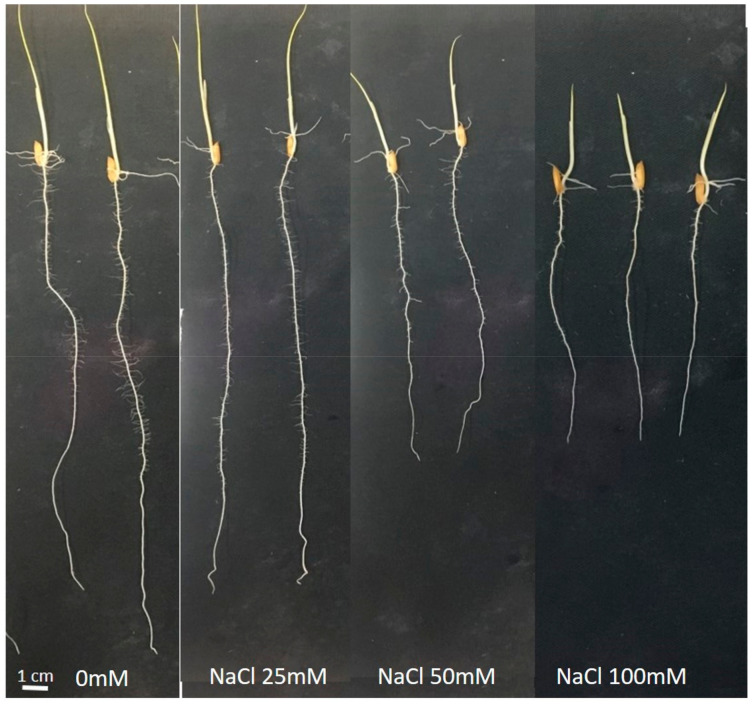
Seedlings of *Oryza sativa* cv. INCA LP-5 grown for 7 days in germination paper rolls at 28 to 30 °C with one of four salt concentrations (0, 25, 50 and 100 mM NaCl).

Although it is not possible to compare both experiments directly, the salt stress during the germination period and seedling stage of seven days in the roll experiment caused less reduction in biomass and NL than in the rhizotron experiment with a duration of 21 days. In the rolls experiment, only the biomass of LP-5 was reduced by salt stress (Table 2). Furthermore, the reductions in NL were about 15% (Perla) and 50% (LP-5), much lower than in the rhizotrons experiment (see above). In a preliminary experiment, a lower salt concentration of 25 mM did not show significant effects during seven days of growth in paper rolls (Figure A2).

Rice is considered salt-sensitive, especially during the early vegetation stage and reproductive stages [10,46,59,60], but relatively tolerant to salt stress during germination [2], which could explain the stronger salt effect in the rhizotron experiment. The germination rate was not reduced at this level of salt stress in our experiments (data not shown). Higher levels of salinity from 5 to 10 and 15 dS m^−1^ can also decrease the germination rate of rice seeds as shown by [12] until a germination rate of nearly zero at 20 dS m^−1^ [61]. Growth responses to salinity are the reactions to two different effects [62]: first, the osmotic effect of the rooting medium could have reduced growth in the rolls experiment, whereas second, in the rhizotron experiment with longer growing time for the plants, the effect of salt toxicity inside the plant have probably led to strong growth impairments.

The results of our study show that mainly the size-related and extent-related root characteristics were affected by salt (exception average root width), and roughly 70% of the variance of these characteristics could be explained by NaCl (Figure 5). This applied to both genotypes, although the network length in LP-5 was almost twice as long as in Perla. Reductions in network length under salinity were found in a wide range of species including *Oryza* spp. [26,45,63], which is linked to suppressed root cell division and elongation [64].

The ability of plants to change their root morphology in response to environmental conditions is known as root plasticity and plays an important role in plant adaptation to stress conditions [65,66]. However, root plasticity and plant growth are not always linked under stress conditions [65]. Thus, roots and shoots can be affected differently by salt stress. This was confirmed by our rhizotron experiment, where, despite the almost identical effects of salt stress on the network length (reduction about 70%, Table 3), the aboveground biomass was less reduced in Perla than in LP-5 (about 50% vs. 70%, Figure 2a). Consequently, the ratio of root length (m) to shoot fresh weight (g) was reduced under salt stress clearly for Perla (from 23.8 m g^−1^ to 16.4 m g^−1^). For LP-5, this ratio was almost the same in both salt levels (about 31.5 m g^−1^). Usually, increased root-to-shoot ratios are thought to improve the source-to-sink ratio for water and nutrients [67] and could therefore be an asset under salinity. On the other hand, a reduced root-to-shoot ratio could reduce the salt flux to the shoots and may result in an increasing salt tolerance [68]. Considering the lower reduction of shoot biomass due to salt stress for Perla than LP-5, under these experimental conditions, a lower root-to-shoot ratio seems to be beneficial. However, this may not have consequences for advanced stages of growth and the ultimate yield. Higher sensitivity of roots than shoots to salt stress was also shown for eight rice varieties during the early growth stage in another study [12].

Because of the higher sensitivity of roots than shoots to salt stress, root growth can be suggested as an indicator of salinity tolerance and for the determination of a salinity threshold of a particular variety. However, one has to consider that the sensitivity of roots and shoots to salt stress varies with clear differences between plant species and developmental stages [66].

Besides the total network length, the number of fine roots is considered to play a major role in nutrition (especially for P) and water absorption [43]. However, in our study, the root width (ARW) was not affected by salt (Table 3), which can be related to the small diameter of roots with <0.03 mm at this early plant development stage. Further studies with longer experimental time are needed to provide information on root diameter under salinity.

In contrast to the root size, properties related to root distribution and shape were hardly affected by salt stress in our experiments (exception: number of roots, Figure 5). Root branching, especially the development of lateral roots can influence root hydraulic conductivity and water supply [26], which can be important for plants growing under conditions of salinity. However, divergent results exist regarding salt stress affecting the development of lateral roots [25,69], and in accordance with our experiment, the authors of [70] noted that lateral root formation was less affected by salinity than root elongation. Corresponding to their small reaction to salt stress, clear and significant correlations between distribution and shape related properties with shoot biomass weight were rarely found.

### 4.2. Differences between the Genotypes

Under our experimental conditions, clear effects of the cultivars were found. LP-5 had a higher biomass and a larger root system, but was more affected by salt stress than Perla, especially in the roll experiment (Table 2). Often, cultivars with higher biomass were found to be more susceptible, while other cultivars might have saline-tolerant genes, but their productivity level is low [46,71]. Although yield components develop later, the shoot development at early growth stadium is suitable to predict salt tolerance and to screen cultivars, as variations at this stage are genetically controlled [45].

While root-size-related characteristics were found to be larger for LP-5, the network solidity (NS), which describes the total network area related to the convex area, was higher for Perla (Table 3). This means that the root system of Perla grows denser, while the root system of LP-5 is more exploratory, which can be an asset under dry conditions, which LP-5 is adapted to. However, it can be a disadvantage for salty soils as the salt flux to the shoots may be increased. Under salt stress, the network solidity further increased for Perla, which was not found for LP-5. However, to answer the question of whether the denser rooting in the root-system area will finally be an asset for Perla on salty sites, more studies at different development stages are necessary, which was also suggested in a phenotyping study of rice populations by Topp et al. [58].

### 4.3. The Role of P and BR Interrelated to Salt Stress

The effects of the other experimental factors, P level and BRs, were lower than the salt effect and often inconsistent for the different traits (Figure 5). Usually, without salt stress, the effect of P was more pronounced (significant interactive effects of NaCl × P). This became especially true for LP-5, for which the plant dry weight, P uptake and root length were only affected by P if no salt stress was applied. For Perla, however, salt stress and P shortage were additive, since salt stress combined with P deficiency resulted in further reductions in plant dry weight, P uptake and root length, as well as some other root characteristics (Figure 2, Table 3). Conversely, one could conclude that sufficient P supply contributes to the mitigation of salt stress and that this potential is higher for Perla than for LP-5. In nature, salt stress is often related to P deficiency, and P supply was often suggested as a promising approach to improve salt tolerance, as for example shown for phaseolus beans [72]. In studies with maize [22] and barley [30], salinity and P availability also affected the crop shoot growth, but with a more marked effect of P than in our experiment, which, however, can be related to the higher salt tolerance of maize and barley in comparison with rice.

Root length and surface (NL and NSA) are more important for the adaptation to low P levels than the root volume or root diameter (NV or ARW), and root elongation was found under P deficiency for various species, including rice [73,74]. Thus, one could also have assumed that the root length or at least the ratio of root length to shoot weight would have been increased under P deficiency. However, this was not the case in our rhizotron experiment, which might be related to the cultivation in solution and not in soil. Similarly, we could not observe an enhanced formation of lateral roots (RD) or a higher ratio of network width (NW) to network depth (ND) under P deficiency (Table 3) as previously shown in the field. There, when P is limited and mainly available from topsoil layers, root growth might be more directed towards increasing network width than deep-rooting [75,76,77].

Brassinosteroids had hardly any effect on the development of the young plants in our experiments. Only for the P concentration in the LP-5 plants and consequently the P uptake, higher values were measured in the BR treatment of the rhizotron experiment (Figure 2b). As this was only the case in the treatment without salt stress combined with sufficient P, and as the standard deviation was very high, this result should not be over-interpreted. The fact that BR can contribute to P nutrition was shown by Talaat and Shawky [33] for wheat cultivars, but this would rather be relevant under P deficiency.

Similarly, effects of BRs on the root system revealed different results, but clear supporting effects were not observed. Furthermore, it could not be found that the BR treatment mitigates salt stress, which is in contrast to previous studies showing that BR treatments can reduce impacts of salt stress in rice and maize [39,40,78], as also stated in the introduction. These results also contradict those shown by Vázquez-Glaría et al. [79] for these same rice cultivars, where the root length of LP-5 was increased after pre-treatment with BRs as well as the lateral root density of LP-5 and Perla. Obviously, the type of BR and time of application is decisive here. Vázquez-Glaría et al. [79] used the BR analog DI-31 for 30 min and in the present study, seeds were pre-treated with the natural BR 24-epibrassinolide for several hours. Based on that, the differences in both BRs’ structure and pre-treatment time could be a key for success or failure of BR application. Considering the partly opposing results of the roll and the rhizotron experiment, plant development and growing conditions can also be the reason for different outcomes regarding the effect of BR.

## 5. Conclusions

Our results show that root length and related traits are suitable for studying responses of rice cultivars to salt stress during early plant development. However, the effects of salt stress may vary widely depending on growing conditions, rice varieties, duration of salt stress and stadium of plant development. Therefore, further studies should be carried out in order to assess the importance of early plant development on later growth stages and rice yield. Mitigation of salt stress by addition of P and BRs was not pronounced and seems to be dependent on the cultivar.

## Figures and Tables

**Table 1:** Root-system traits used for root analysis in mini-rhizotrons with categories after Topp et al. (2013), abbreviation and unit, analyzed with GiA Roots (Galkovskyi et al. 2012) and the ImageJ plugin SmartRoot (lateral root density, root angle; Lobet et al. 2011).

Category	Root trait	Abbreviation	Unit
Size	Network length	NL	cm
	Network volume	NV	cm^3^
	Network surface area	NSA	cm^2^
	Network perimeter	NP	cm
	Network area	NA	cm^2^
	Average root width	ARW	mm
Distribution	Specific root length	SRL	cm cm^−3^
	Network solidity	NS	%
	Network bushiness	NB	n n^−1^
	Lateral root density	RD	n cm^−1^
	Maximum number of roots	MaNR	n
	Median number of roots	MeNR	n
	Network length distribution	NLD	n n^−1^
Extent	Network convex area	NCA	cm^2^
	Major ellipse axis	MaEA	cm
	Minor ellipse axis	MiEA	cm
	Network depth	ND	cm
	Network width	NW	cm
Shape	Ellipse axis ratio	EAR	cm cm^−1^
	Network width-to-depth ratio	NWDR	cm cm^−1^
	Root angle	RA	°

**Table 2:** Root parameters of the two rice cultivars INCA LP-5 and Perla de Cuba after seven days of growth in cigar rolls with or without salt (NaCl) and 24-epibrassinolide (BR) pre-treatment (mean ± SD). Significance of factors NaCl, BR and their interactions after ANOVA (*p* < 0.05 * 0.01 ** 0.001 ***) are given for both cultivars. Significant differences between cultivars in each treatment are indicated.

		0 mM NaCl		50 mM NaCl		
		0 M BR	10^−6^ M BR	0 M BR	10^−6^ M BR	Significances
Shoot fresh weight	INCA LP-5	77.7 ± 5.39	72.9 ± 7.21	47.2 ± 8.23	53.7 ± 96.4	NaCl ***
[mg]	Perla de Cuba	51.1 ± 10.6 **	58.9 ± 4.23 **	52.3 ± 15 0	58.9 ± 5.31	
Root fresh weight	INCA LP-5	21.1 ± 2.26	19.5 ± 1.26	11.5 ± 4.40	12.6 ± 3.28	NaCl ***
[mg]	Perla de Cuba	16.1 ± 6.11	14.8 ± 5.23	16.3 ± 7.52	13.8 ± 4.06	
Root-to-shoot ratio	INCA LP-5	0.27 ± 0.03	0.27 ± 0.04	0.24 ± 0.05	0.23 ± 0.04	
[mg mg^−1^]	Perla de Cuba	0.31 ± 0.10	0.25 ± 0.09	0.30 ± 0.07	0.23 ± 0.06	
Primary root length	INCA LP-5	19.0 ± 3.47	19.7 ± 4.01	13.3 ± 6.65	12.5 ± 6.52	NaCl ***
[cm]	Perla de Cuba	13.5 ± 4.46 ***	9.67 ± 4.06 ***	15.8 ± 7.51	12.8 ± 5.86	BR *
Lateral root density	INCA LP-5	14.4 ± 2.06	11.8 ± 1.53	10.8 ± 3.95	11.7 ± 2.02	NaCl **, BR *
[cm^−1^]	Perla de Cuba	14.5 ± 1.96	14.2 ± 1.51 ***	12.2 ± 2.46	11.5 ± 2.03	NaCl ***
Network length	INCA LP-5	80.6 ± 25.6	75.7 ± 19.4	41.1 ± 33.5	33.5 ± 18.8	NaCl ***
[cm]	Perla de Cuba	61.5 ± 25.9	42.1 ± 20.0 ***	51.0 ± 28.2	34.9 ± 18.1	BR **

**Table 3:** Selected root-system traits of the two rice genotypes INCA LP-5 and Perla de Cuba after three weeks of growth in mini-rhizotrons (mean ± SD). Significant differences between treatments of phosphorus (P), salt (NaCl), 24-epibrassinolide (BR) and their interactions after ANOVA are shown by asterisks (*p* < 0.05 * 0.01 ** 0.001 ***) and given for both cultivars. Significant differences between cultivars in each treatment are indicated. NL: network length; ARW: average root width; NS: network solidity; NB: network bushiness; RD: lateral root density; MaNR: maximum number of roots; NLD: network length distribution; NCA: network convex area; ND: network depth; NW: network width; NWDR: network width-to-depth ratio; RA: root angle.

		0 mM NaCl				50 mM NaCl				
		10 ppm P		1 ppm P		10 ppm P		1 ppm P		
		0 M BR	10^−6^ M BR	0 M BR	10^−6^ M BR	0 M BR	10^−6^ M BR	0 M BR	10^−6^ M BR	Significance
NL	INCA LP-5	756 ± 241	779 ± 33.6	466 ± 134	388 ± 148	224 ± 135	152 ± 91.4	150 ± 67.3	204 ± 117	P ***, NaCl ***, P × NaCl ***
[cm]	Perla de Cuba	420 ± 34.3 *	378 ± 115 ***	286 ± 51.1 *	310 ± 73.1	128 ± 18.6	74.5 ± 35.4	74.5 ± 60.6	94.4 ± 60.2 *	P **, NaCl ***, P × NaCl *
ARW	INCA LP-5	0.18 ± 0.00	0.18 ± 0.00	0.18 ± 0.00	0.19 ± 0.00	0.18 ± 0.00	0.18 ± 0.01	0.19 ± 0.00	0.18 ± 0.00	P *
[mm]	Perla de Cuba	0.18 ± 0.00	0.19 ± 0.00	0.18 ± 0.00	0.18 ± 0.00 *	0.19 ± 0.01	0.18 ± 0.00	0.18 ± 0.00	0.18 ± 0.00	NaCl × BR *
NS	INCA LP-5	8.66 ± 1.09	8.79 ± 1.31	7.39 ± 1.42	7.09 ± 1.27	6.64 ± 1.46	7.71 ± 2.20	7.81 ± 2.29	9.17 ± 1.54	P × NaCl *
[%]	Perla de Cuba	9.70 ± 1.51	9.31 ± 1.36	9.35 ± 2.33	10.2 ± 1.53 **	10.2 ± 1.47 **	11.2 ± 2.29 *	12.5 ± 3.26 *	11.7 ± 1.94	NaCl *
NB	INCA LP-5	2.15 ± 0.53	2.27 ± 0.13	2.22 ± 0.52	2.38 ± 1.19	2.65 ± 0.54	2.97 ± 0.76	2.35 ± 0.37	2.21 ± 0.34	ns
[n n^−1^]	Perla de Cuba	3.03 ± 1.45	4.00 ± 0.91 **	3.20 ± 1.10	3.32 ± 1.11	2.54 ± 0.98	2.05 ± 0.58	2.27 ± 1.01	1.82 ± 0.39	NaCl ***
RD	INCA LP-5	15.3 ± 2.71	16.8 ± 2.60	14.4 ± 1.68	13.5 ± 1.58	13.1 ± 2.10	14.8 ± 2.22	13.5 ± 1.61	12.5 ± 2.06	P *, NaCl *
[n cm^−1^]	Perla de Cuba	14.4 ± 2.26	14.2 ± 1.47	11.9 ± 1.30 *	10.4 ± 1.75 *	11.9 ± 2.77	11.7 ± 1.07 *	11.8 ± 2.59	12.3 ± 2.68	P *, P *NaCl *
MaNR	INCA LP-5	49.6 ± 13.5	48.6 ± 10.8	31.2 ± 9.76	24.2 ± 6.18	18.4 ± 9.74	17.2 ± 6.94	15.2 ± 4.76	20.0 ± 5.48	P ***, NaCl ***, P × NaCl ***
[n]	Perla de Cuba	46.6 ± 5.18	42.6 ± 13.9	37.2 ± 8.26	47.8 ± 11.4 **	28.4 ± 7.13	15.6 ± 3.21	16.4 ± 9.18	21.8 ± 10.4	NaCl ***, PxBR **
NLD	INCA LP-5	0.91 ± 0.46	1.17 ± 0.46	1.06 ± 0.46	1.24 ± 0.50	1.38 ± 0.55	1.52 ± 0.07	0.90 ± 0.31	1.51 ± 0.34	BR *
[n n^−1^]	Perla de Cuba	1.14 ± 0.92	0.72 ± 0.30	1.22 ± 0.59	0.66 ± 0.53	0.11 ± 0.14 **	0.33 ± 0.40 ***	0.67 ± 0.43	0.16 ± 0.25 ***	NaCl ***
NCA	INCA LP-5	111 ± 35.2	113 ± 19.2	82.3 ± 27.8	73.1 ± 26.4	43.2 ± 22.6	30.2 ± 22.2	28.3 ± 13.5	31.4 ± 23.1	P *, NaCl ***
[cm^2^]	Perla de Cuba	54.1 ± 5.55 **	52.3 ± 11.3 ***	41.1 ± 13.6 *	39.0 ± 13.6 *	17.1 ± 2.46 *	9.18 ± 4.78	9.63 ± 9.50 *	9.81 ± 5.68	P **, NaCl ***
ND	INCA LP-5	16.0 ± 1.49	17.0 ± 2.64	16.1 ± 2.39	15.8 ± 2.69	13.8 ± 2.96	9.85 ± 4.09	11.2 ± 2.71	10.2 ± 3.87	NaCl ***
[cm]	Perla de Cuba	11.7 ± 2.07 **	13.0 ± 1.15 *	10.5 ± 2.34 **	9.41 ± 2.47 **	6.25 ± 0.74 **	5.64 ± 2.59	4.59 ± 2.14 **	5.88 ± 1.84	P *, NaCl ***
NW	INCA LP-5	10.7 ± 2.30	10.4 ± 2.95	8.40 ± 3.09	6.11 ± 1.47	5.32 ± 2.27	3.81 ± 2.45	3.84 ± 1.39	3.54 ± 1.33	P *, NaCl ***
[cm]	Perla de Cuba	8.28 ± 1.11	7.25 ± 1.23	6.23 ± 1.10	6.56 ± 1.33	5.66 ± 1.11	2.76 ± 1.10	2.43 ± 1.68	3.06 ± 1.31	P **, NaCl ***, P × BR **
NWDR	INCA LP-5	0.67 ± 0.15	0.64 ± 0.27	0.53 ± 0.18	0.39 ± 0.08	0.39 ± 0.15	0.35 ± 0.13	0.33 ± 0.07	0.35 ± 0.02	P *, NaCl ***
[cm cm^−1^]	Perla de Cuba	0.73 ± 0.14	0.56 ± 0.09	0.60 ± 0.09	0.72 ± 0.19 **	0.91 ± 0.18 **	0.54 ± 0.20	0.52 ± 0.32	0.58 ± 0.35	P × BR *
RA	INCA LP-5	109 ± 31.0	95.1 ± 11.6	103 ± 29.5	82.7 ± 10.5	76.6 ± 19.1	51.6 ± 49.9	57.3 ± 32.3	64.0 ± 7.16	NaCl ***
[°]	Perla de Cuba	112 ± 13.3	107 ± 14.3	106 ± 13.7	103 ± 17.4	103 ± 15.4 *	50.6 ± 37.2	31.3 ± 45.3	51.7 ± 32.7	NaCl ***, P *, P × BR *, P × NaCl × BR *

## Data Availability

The data that support the findings of this study are available from the corresponding author upon reasonable request.

## Appendix A

Table A1: Root-system traits, Figure A1: Phosphorus uptake, Figure A2: Seedlings of *Oryza sativa* cv. INCA LP-5.

**Table A1:** Root-system traits of the two rice genotypes INCA LP-5 and Perla de Cuba after three weeks of growth in mini-rhizotrons (mean ± SD). Significant differences between treatments of phosphorus (P), salt (NaCl), 24-epibrassinolide (BR) and their interactions after ANOVA are shown by asterisks (*p* < 0.05 * 0.01 ** 0.001 ***; *p* > 0.05 ns). NL: network length; NV: network volume; NSA: network surface area; NP: network perimeter; NA: network area; ARW: average root width; SRL: specific root length; NS: network solidity; NB: network bushiness; RD: lateral root density; MaNR: maximum number of roots; MeNR: median number of roots; NLD: network length distribution; NCA: network convex area; MaEA: major ellipse axis; MiEA: minor ellipse axis; ND: network depth; NW: network width; EAR: ellipse axis ratio; NWDR: network width-to-depth ratio; RA: root angle.

		0 mM NaCl				50 mM NaCl				
		10 ppm P		1 ppm P		10 ppm P		1 ppm P		
		0 M BR	10^−6^ M BR	0 M BR	10^−6^ M BR	0 M BR	10^−6^ M BR	0 M BR	10^−6^ M BR	Significance
NL	INCA LP-5	756 ± 241	779 ± 33.6	466 ± 134	388 ± 148	224 ± 135	152 ± 91.4	150 ± 67.3	204 ± 117	P ***, NaCl ***, P × NaCl ***
[cm]	Perla de Cuba	420 ± 34.3	378 ± 115	286 ± 51.1	310 ± 73.1	128 ± 18.6	74.5 ± 35.4	74.5 ± 60.6	94.4 ± 60.2	P **, NaCl ***, P × NaCl *
NV	INCA LP-5	0.21 ± 0.06	0.21 ± 0.01	0.13 ± 0.03	0.11 ± 0.04	0.06 ± 0.04	0.04 ± 0.03	0.04 ± 0.02	0.06 ± 0.03	P ***, NaCl ***, P × NaCl **
[cm^3^]	Perla de Cuba	0.11 ± 0.01	0.11 ± 0.03	0.08 ± 0.02	0.09 ± 0.02	0.04 ± 0.01	0.02 ± 0.01	0.02 ± 0.02	0.03 ± 0.02	P **, NaCl ***
NSA	INCA LP-5	43.3 ± 13.6	44.6 ± 2.13	26.8 ± 7.32	22.8 ± 8.61	13.0 ± 7.88	8.77 ± 5.35	8.81 ± 4.01	11.8 ± 6.82	P ***, NaCl ***, P × NaCl ***
[cm^2^]	Perla de Cuba	23.9 ± 2.06	21.9 ± 6.33	16.6 ± 3.09	17.8 ± 4.25	7.59 ± 1.19	4.31 ± 2.07	4.32 ± 3.54	5.42 ± 3.48	P **, NaCl ***, P × NaCl *
NP	INCA LP-5	1084 ± 346	1111 ± 56.1	665 ± 179	575 ± 214	327 ± 203	221 ± 136	224 ± 100	297 ± 173	P ***, NaCl ***, P × NaCl ***
[cm]	Perla de Cuba	595 ± 57.5	554 ± 155	413 ± 79.0	440 ± 103	195 ± 32.9	110 ± 51.7	111 ± 90.3	136 ± 86.5	P **, NaCl ***, P × NaCl *
NA	INCA LP-5	9.49 ± 3.00	9.72 ± 0.53	5.86 ± 1.51	5.12 ± 1.90	2.90 ± 1.80	1.94 ± 1.21	1.99 ± 0.91	2.62 ± 1.52	P ***, NaCl ***, P × NaCl **
[cm^2^]	Perla de Cuba	5.19 ± 0.51	4.89 ± 1.32	3.65 ± 0.72	3.87 ± 0.92	1.74 ± 0.31	0.96 ± 0.46	0.97 ± 0.80	1.19 ± 0.77	P **, NaCl ***, P × NaCl *
ARW	INCA LP-5	0.18 ± 0.00	0.18 ± 0.00	0.18 ± 0.00	0.19 ± 0.00	0.18 ± 0.00	0.18 ± 0.01	0.19 ± 0.00	0.18 ± 0.00	P *
[mm]	Perla de Cuba	0.18 ± 0.00	0.19 ± 0.00	0.18 ± 0.00	0.18 ± 0.00	0.19 ± 0.01	0.18 ± 0.00	0.18 ± 0.00	0.18 ± 0.00	NaCl × BR *
SRL	INCA LP-5	3670 ± 144	3687 ± 75.7	3595 ± 177	3430 ± 121	3581 ± 153	3689 ± 307	3481 ± 168	3623 ± 216	P *
[cm cm^3^]	Perla de Cuba	3717 ± 57.8	3542 ± 184	3585 ± 90.3	3628 ± 92.5	3405 ± 232	3590 ± 134	3597 ± 148	3687 ± 196	NaClxBR *
NS	INCA LP-5	8.66 ± 1.09	8.79 ± 1.31	7.39 ± 1.42	7.09 ± 1.27	6.64 ± 1.46	7.71 ± 2.20	7.81 ± 2.29	9.17 ± 1.54	PxNaCl *
[%]	Perla de Cuba	9.70 ± 1.51	9.31 ± 1.36	9.35 ± 2.33	10.2 ± 1.53	10.2 ± 1.47	11.2 ± 2.29	12.5 ± 3.26	11.7 ± 1.94	NaCl *
NB	INCA LP-5	2.15 ± 0.53	2.27 ± 0.13	2.22 ± 0.52	2.38 ± 1.19	2.65 ± 0.54	2.97 ± 0.76	2.35 ± 0.37	2.21 ± 0.34	ns
[n n^−1^]	Perla de Cuba	3.03 ± 1.45	4.00 ± 0.91	3.20 ± 1.10	3.32 ± 1.11	2.54 ± 0.98	2.05 ± 0.58	2.27 ± 1.01	1.82 ± 0.39	NaCl ***
RD	INCA LP-5	15.3 ± 2.71	16.8 ± 2.60	14.4 ± 1.68	13.5 ± 1.58	13.1 ± 2.10	14.8 ± 2.22	13.5 ± 1.61	12.5 ± 2.06	P *, NaCl *
[n cm^−1^]	Perla de Cuba	14.4 ± 2.26	14.2 ± 1.47	11.9 ± 1.30	10.4 ± 1.75	11.9 ± 2.77	11.7 ± 1.07	11.8 ± 2.59	12.3 ± 2.68	P *, P *, NaCl *
MaNR	INCA LP-5	49.6 ± 13.5	48.6 ± 10.8	31.2 ± 9.76	24.2 ± 6.18	18.4 ± 9.74	17.2 ± 6.94	15.2 ± 4.76	20.0 ± 5.48	P ***, NaCl ***, P × NaCl ***
[n]	Perla de Cuba	46.6 ± 5.18	42.6 ± 13.9	37.2 ± 8.26	47.8 ± 11.4	28.4 ± 7.13	15.6 ± 3.21	16.4 ± 9.18	21.8 ± 10.4	NaCl ***, P × BR **
MeNR	INCA LP-5	23.6 ± 7.50	21.4 ± 4.62	14.8 ± 5.93	12.4 ± 7.40	7.00 ± 3.54	5.80 ± 2.39	6.80 ± 2.95	9.00 ± 1.83	P *, NaCl ***, P × NaCl **
[n]	Perla de Cuba	18.8 ± 10.1	10.6 ± 2.88	12.6 ± 4.93	15.8 ± 6.30	12.2 ± 4.32	8.00 ± 2.55	7.80 ± 4.82	11.8 ± 5.22	NaCl *, P × BR **
NLD	INCA LP-5	0.91 ± 0.46	1.17 ± 0.46	1.06 ± 0.46	1.24 ± 0.50	1.38 ± 0.55	1.52 ± 0.07	0.90 ± 0.31	1.51 ± 0.34	BR *
[n n^−1^]	Perla de Cuba	1.14 ± 0.92	0.72 ± 0.30	1.22 ± 0.59	0.66 ± 0.53	0.11 ± 0.14	0.33 ± 0.40	0.67 ± 0.43	0.16 ± 0.25	NaCl ***
NCA	INCA LP-5	111 ± 35.2	113 ± 19.2	82.3 ± 27.8	73.1 ± 26.4	43.2 ± 22.6	30.2 ± 22.2	28.3 ± 13.5	31.4 ± 23.1	P *, NaCl ***
[cm^2^]	Perla de Cuba	54.1 ± 5.55	52.3 ± 11.3	41.1 ± 13.6	39.0 ± 13.6	17.1 ± 2.46	9.18 ± 4.78	9.63 ± 9.50	9.81 ± 5.68	P **, NaCl ***
MaEA	INCA LP-5	13.6 ± 1.55	13.7 ± 1.96	13.4 ± 1.62	13.8 ± 3.28	11.5 ± 2.78	8.72 ± 3.22	9.19 ± 2.42	9.91 ± 3.93	NaCl ***
[cm]	Perla de Cuba	8.74 ± 1.36	8.57 ± 1.52	7.77 ± 1.54	7.91 ± 1.79	5.40 ± 0.58	4.27 ± 1.66	3.98 ± 1.73	4.50 ± 1.39	NaCl ***
MiEA	INCA LP-5	7.40 ± 2.20	7.35 ± 1.40	6.44 ± 1.92	4.97 ± 1.18	3.98 ± 2.13	3.45 ± 2.01	2.93 ± 1.16	2.78 ± 1.10	P *, NaCl ***
[cm]	Perla de Cuba	5.77 ± 0.26	5.85 ± 0.83	4.83 ± 1.07	4.53 ± 0.62	2.86 ± 0.32	2.17 ± 0.78	1.84 ± 1.07	2.19 ± 0.78	P **, NaCl ***
ND	INCA LP-5	16.0 ± 1.49	17.0 ± 2.64	16.1 ± 2.39	15.8 ± 2.69	13.8 ± 2.96	9.85 ± 4.09	11.2 ± 2.71	10.2 ± 3.87	NaCl ***
[cm]	Perla de Cuba	11.7 ± 2.07	13.0 ± 1.15	10.5 ± 2.34	9.41 ± 2.47	6.25 ± 0.74	5.64 ± 2.59	4.59 ± 2.14	5.88 ± 1.84	P *, NaCl ***
NW	INCA LP-5	10.7 ± 2.30	10.4 ± 2.95	8.40 ± 3.09	6.11 ± 1.47	5.32 ± 2.27	3.81 ± 2.45	3.84 ± 1.39	3.54 ± 1.33	P *, NaCl ***
[cm]	Perla de Cuba	8.28 ± 1.11	7.25 ± 1.23	6.23 ± 1.10	6.56 ± 1.33	5.66 ± 1.11	2.76 ± 1.10	2.43 ± 1.68	3.06 ± 1.31	P **, NaCl ***, P × BR **
EAR	INCA LP-5	0.55 ± 0.16	0.55 ± 0.19	0.49 ± 0.16	0.37 ± 0.07	0.36 ± 0.21	0.37 ± 0.13	0.31 ± 0.08	0.28 ± 0.05	NaCl **
[cm cm^−1^]	Perla de Cuba	0.68 ± 0.12	0.70 ± 0.15	0.64 ± 0.16	0.59 ± 0.10	0.54 ± 0.09	0.54 ± 0.16	0.44 ± 0.13	0.53 ± 0.25	NaCl **
NWDR	INCA LP-5	0.67 ± 0.15	0.64 ± 0.27	0.53 ± 0.18	0.39 ± 0.08	0.39 ± 0.15	0.35 ± 0.13	0.33 ± 0.07	0.35 ± 0.02	P *, NaCl ***
[cm cm^−1^]	Perla de Cuba	0.73 ± 0.14	0.56 ± 0.09	0.60 ± 0.09	0.72 ± 0.19	0.91 ± 0.18	0.54 ± 0.20	0.52 ± 0.32	0.58 ± 0.35	PxBR *
RA	INCA LP-5	109 ± 31.0	95.1 ± 11.6	103 ± 29.5	82.7 ± 10.5	76.6 ± 19.1	51.6 ± 49.9	57.3 ± 32.3	64.0 ± 7.16	NaCl ***
[°]	Perla de Cuba	112 ± 13.3	107 ± 14.3	106 ± 13.7	103 ± 17.4	103 ± 15.4	50.6 ± 37.2	31.3 ± 45.3	51.7 ± 32.7	NaCl ***, P *, P × BR *, P × NaCl × BR *
